# A Manual of Diseases of the Eye

**Published:** 1903-08

**Authors:** 


A Manual of Diseases of the Eye. For Students and General Practitioners. By Clarence A. Veasey, A.M., M. D., Demonstrator of Ophthalmology in Jefferson Medical College, Philadelphia. Duodecimo, 412 pages, with 194 engravings and 10 full-page colored plates. Lea Brothers & Co., Philadelphia and New York. 1903. (Price, $2.00 net.)

This excellent manual is one of the best that has been issued in recent years. For students and practitioners, it is an ideal handbook of reference, being practical, concise, copiously illustrated and showing the excellent judgment of the author in choosing what to include and what to omit. The therapeutics do not embrace some of the newer standard drugs, and the suggestions in treatment in the more serious diseases are rather vague; but aside from this no fault can be found and the book should be in the library of every physician wishing a work on opthalmology for reference.

